# Biogenic Synthesis of Metal Nanoparticles Using a Biosurfactant Extracted from Corn and Their Antimicrobial Properties

**DOI:** 10.3390/nano7060139

**Published:** 2017-06-06

**Authors:** Sergio Gómez-Graña, María Perez-Ameneiro, Xanel Vecino, Isabel Pastoriza-Santos, Jorge Perez-Juste, José Manuel Cruz, Ana Belén Moldes

**Affiliations:** 1 Departamento de Química Física CINBIO, Universidade de Vigo, 36310 Vigo, Spain; pastoriza@uvigo.es (I.P.-S.); juste@uvigo.es (J.P.-J.); 2 Chemical Engineering Department, School of Industrial Engineering (EEI), University of Vigo, Campus As Lagoas-Marcosende, 36310 Vigo-Pontevedra, Spain; mp.ameneiro@uvigo.es (M.P.-A.); xanel.vecino@uvigo.es (X.V.); jmcruz@uvigo.es (J.M.C.); amoldes@uvigo.es (A.B.M.); 3 CEB-Centre of Biological Engineering, University of Minho, Campus de Gualtar, 4710-057 Braga, Portugal

**Keywords:** biosurfactant, corn stream, silver NPs, gold NPs, green synthesis

## Abstract

A new and promising biosurfactant extracted from corn steep liquor has been used for the green synthesis of gold and silver nanoparticles (NPs) in a one-step procedure induced by temperature. Most of the biosurfactants proposed in the literature are produced by pathogenic microorganisms; whereas the biosurfactant used in the current work was extracted from a liquid stream, fermented spontaneously by lactic acid bacteria, which are “generally recognized as safe” (GRAS) microorganisms. The reduction of a gold precursor in the presence of a biosurfactant gives rise to a mixture of nanospheres and nanoplates with distinct optical features. Moreover, the growth of nanoplates can be promoted by increasing the reaction temperature to 60 °C. In the case of silver, the biosurfactant just induces the formation of pseudo-spherical NPs. The biosurfactant plays a key role in the reduction of the metal precursor, as well as in the stabilization of the resulting NPs. Furthermore, the antimicrobial activity of the resulting silver colloids has been analyzed against *Escherichia coli*, *Pseudomonas aeruginosa* and *Staphylococcus aureus*. The biosurfactant stabilized NPs slightly increased the inhibition of *E. coli* in comparison with citrate stabilized Ag NPs. The use of this biosurfactant extracted from corn steep liquor for the synthesis of metal NPs contributes to enhancing the application of green technologies and increasing the utilization of clean, non-toxic and environmentally safe production processes. Therefore, it can help to reduce environmental impact, minimize waste and increase energy efficiency in the field of nanomaterials.

## 1. Introduction

One of the fields that has witnessed spectacular growth in recent decades has been that of nanotechnology. Such interest is due largely to its potential applications in almost all disciplines of science and technology, from electronics, sensing, and catalysis to cosmetics or drug delivery [1,2,3,4]. Among different nanomaterials, plasmonic nanoparticles (NPs)—mainly gold (Au NPs) and silver (Ag NPs) NPs—have also attracted tremendous attention due to their unique optical properties. Thus, Ag and Au NPs are used in a wide range of applications in different fields like optics (for metal-enhanced fluorescence and surface-enhanced Raman scattering) [5], electronics [6], catalysis [7], sensing [8,9] and biomedicine [10]. 

The optical properties of Au or Ag NPs can be explained in terms of localized surface plasmons. The interaction of the electromagnetic field of incoming light with a nanoparticle leads to the oscillation in phase of its conduction electrons. Such oscillation in phase is the origin of localized surface plasmon resonances (LSPR) [11]. The energy of the LSPR is largely governed by the nature of the material (gold, silver, and copper, among others), the size, the shape and the surrounding medium. For instance, spherical Au NPs between 10 and 100 nm present a LSPR band located approximately between 520 and 570 nm [12], while for spherical Ag NPs the LSPR is located between 390 and 430 nm [13]. Additionally, the shape of the nanoparticle could also influence its optical properties. Thus, for instance, gold nanoprisms display at least two well-defined LSPR bands ascribed to the in-plane and out-of-plane resonances that can be tuned into the NIR region of the spectrum by controlling the length and the thickness of the nanoprisms [14]. In the literature, a wide range of protocols to synthetize Ag and Au NPs with defined shape, size and surface chemistry can be found, such as polyol synthesis [15], citrate reduction [12,13], and photoreduction [16], among others. Nevertheless, all these methods usually involve the use of hazardous reactants, which could have numerous harmful effects on the environment and human health. For this reason, the further use of the NPs often requires a purification step to eliminate any hazardous reactants from their synthesis.

Green chemistry has emerged as a novel alternative route for the synthesis of metallic NPs [17,18]. A green synthesis process should not involve the use of any toxic chemicals. Additionally, it should be cost-effective, environmentally friendly and be a zero energy-based, less time-consuming process. Moreover, it should not require the use of any kind of hazardous products. Recent studies have pointed out the use of biosurfactants as an alternative for the synthesis of noble metal NPs, envisaging great potential in the green chemistry field [19,20]. For instance, Reddy et al. have used surfactin, a lipopeptide biosurfactant, as a template and stabilizing agent in the synthesis of noble metal NPs [21,22]. 

Biosurfactants can be produced by different strains of bacteria [23], yeast [24] or fungi [25], being the majority of these biosurfactants produced extracellularly, as well as plant extracts [18] or waste materials [26]. Nevertheless, some microorganisms like *Lactobacillus* strains produce cell-bound biosurfactants [27]. Some of the biosurfactants proposed in the literature are produced by pathogenic microorganisms, reducing their applicability in the synthesis of NPs. For instance, Farias and co-workers have proposed a biosurfactant produced by *Pseudomonas aeruginosa* (a microorganism not listed as “generally recognized as safe”, GRAS), cultivated in a low-cost medium, to stabilize AgNPs obtained via reduction of a silver precursor using NaBH_4_ [28]. Other authors have also reported the use of rhamnolipids, also produced by *P. aeruginosa*, to synthesize silver NPs [29]. In contrast, the US Food and Drug Administration (FDA) have included the lactic acid bacteria in the list of GRAS microorganisms. Therefore, biosurfactants produced by lactic acid bacteria can be considered as non-toxic. Vecino and coworkers [30] have recently found that corn steep liquor (CSL) contains important concentrations of natural biosurfactant, 12.0 ± 0.5 g/Kg of CSL, which is probably produced, as a secondary metabolite, by the lactic acid bacteria that grow spontaneously in the CSL and prevent the growth of pathogenic microorganisms. Taking this into account, the aim of the present work is to exploit the use of the lipopeptide biosurfactant extracted from CSL for the synthesis of Au and Ag NPs in a “one-step” green protocol. To the best of our knowledge, to date, there are no studies reporting the application of this biosurfactant in the synthesis of noble metal NPs. Furthermore, the Au and Ag NPs obtained have been characterized by UV-Vis-NIR spectroscopy, Transmission Electron Microscopy (TEM), and the antimicrobial activity of Ag NPs has also been tested.

## 2. Results and Discussion

### 2.1. Biosurfactant Extraction and Characterization

**Figure 1:**
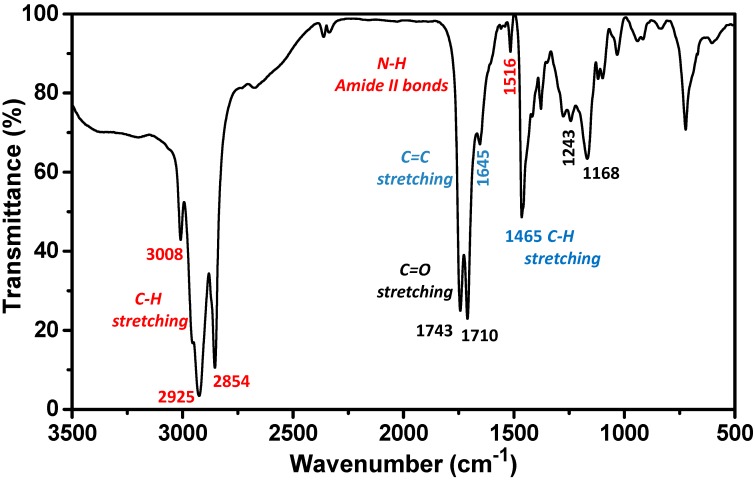
Fourier Transform infrared spectroscopy (FTIR) spectrum of the lipopeptide biosurfactant extracted from corn steep liquor. The color code indicates the assignment of the different bands.

The biosurfactant produced by spontaneous fermentation of lactic acid bacteria on CSL was extracted by using the protocol recently improved by Vecino and co-workers [30]. After a filtration step to remove some large insoluble molecules, the extract was characterized by Fourier Transform infrared spectroscopy (FTIR) spectroscopy. As shown in Figure 1, FTIR spectrum of the biosurfactant indicates the presence of protein-related weak bands. Thus, the broad band at 3200–3550 cm^−1^ can be attributed to O–H stretching vibrations, due to the presence of sugars and fatty acids in the biosurfactant composition. The strong and broad bands at 3000–2850 cm^−1^ and 1465–1375 cm^−1^ denote the presence of C–H stretching, corresponding to CH_2_ and CH_3_ groups of aliphatic chains. Moreover, the C=O bond exhibits two strong peaks at 1743–700 cm^−1^ (corresponding to the amide bonds), and the N–H bond has a weak band at 1516 cm^−1^ (amides and amines bonds). The bands at 1276 and 1743 cm^−1^ are stretching vibrations of C–O and C=O bonds in carboxyl esters. Finally, the bands at 1168, 1243 cm^−1^ 1645 cm^−1^ indicate the presence of fatty acids. Therefore the FTIR analysis confirmed that the extracted biosurfactant comprises lipids and proteins showing a good agreement with the previously reported [30,31]. 

Taking into account that a substance can be considered a biosurfactant when it has the ability to reduce the surface tension of water by at least 8 units [32], we studied the capability of our extracted material as a biosurfactant. It has been previously reported that the CSL biosurfactant is able to reduce the surface tension of water from 72 to 42 mN/m, with a critical micelle concentration of 150 mg/L. On the other hand, the biochemical analysis revealed that the biosurfactant extracted from CSL is composed by 21.9% of proteins, 64.2% of lipids, 5% of sugars and 9% of other components. Moreover, the fatty acids were composed mainly of C16 and C18 fatty acids containing linoelaidic acid, oleic or/and elaidic acid, stearic acid and palmitic acid [31]. Table 1 summarizes the fatty acid compositions and their relative abundance. The fatty acid composition of the biosurfactant extracted from corn steep liquor is quite similar to the composition of surfactin, a very powerful surfactant used as antibiotic [33]. Finally, the composition of the CSL biosurfactant reveals the presence of phytochemicals which are able to reduce metal precursor as well as to provide stability to NPs, as previously reported [17,34,35]. 

In summary, considering that CSL is a byproduct of the corn wet-milling industry, it can be considered as a source of an inexpensive and eco-friendly biosurfactant. Thus, this biosurfactant could find applications in different fields such as pharmaceutical and biomedical science, or cosmetics as well as in food and agricultural science [36,37,38]. Additionally, this type of eco-friendly material could also be categorized as sources for “green synthesis” of noble metal NPs, due to the presence of phytochemicals that are responsible for the reduction of metal salt precursors, as well as their further stabilization [17,34,35]. 

### 2.2. Synthesis of Au NPs Using the Biosurfactant Extracted from CSL

**Figure 2:**
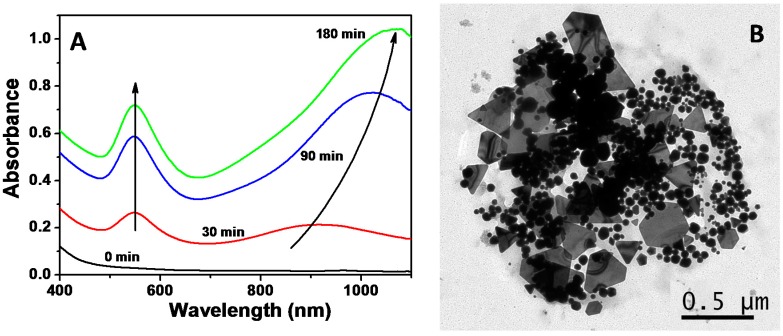
(**A**) Time evolution of visible-NIR spectra during the synthesis of AuNPs in the presence of the biosurfactant extracted from corn steep liquor (CSL) at 60 °C. (**B**) Representative transmission electron microscopy (TEM) image of the synthetized nanoparticles (NPs).

In order to synthesize Au NPs, an aqueous solution of tetrachloroauric acid was mixed with a biosurfactant solution under magnetic stirring and the mixture was heated to 60 °C (see details in the Materials and Methods section). The initial mixture, light yellow due to the presence of Au^3+^ in the solution, evolved to colorless within a few minutes due to the reduction of gold ions, from Au^3+^ to Au*+* [3], and eventually turned wine-red, indicating the reduction of Au^+^ and eventually the formation of Au NPs. This process was followed by Vis-NIR absorption spectroscopy as shown in Figure 2A. While the absorption spectrum of the initial gold salt solution does not present any peaks in the Vis-NIR, as the temperature increases, two LSPR bands appear at 550 nm and 900 nm, indicating the reduction of the gold precursor and the formation of Au NPs. The band at longer wavelengths can be ascribed to non-spherical NPs. As the reaction proceeds, both bands increase in intensity. Additionally, the band initially located at 900 nm red-shifts up to 1050 nm (see Figure 2A). This spectral evolution could be ascribed to either the formation of new particles or to the catalytically reduction of the gold salt on the initially-formed gold seeds mediated by the biosurfactant [39]. After three hours, the optical properties of the dispersion remained unchanged, thus indicating that the reaction was finished. The optical properties remained unaltered for months, indicating the formation of a stable colloidal dispersion.

In order to understand the optical properties, we have further characterized the as-prepared NPs by transmission electron microscopy (TEM). Figure 2B shows a representative TEM image of the final Au NPs, where two different populations can be clearly distinguished: (i) spherical NPs with an average size of 35–40 nm, responsible of the LSPR band at 550 nm; and (ii) nanoplates, which are responsible for the band at NIR region [11,14]. 

**Figure 3:**
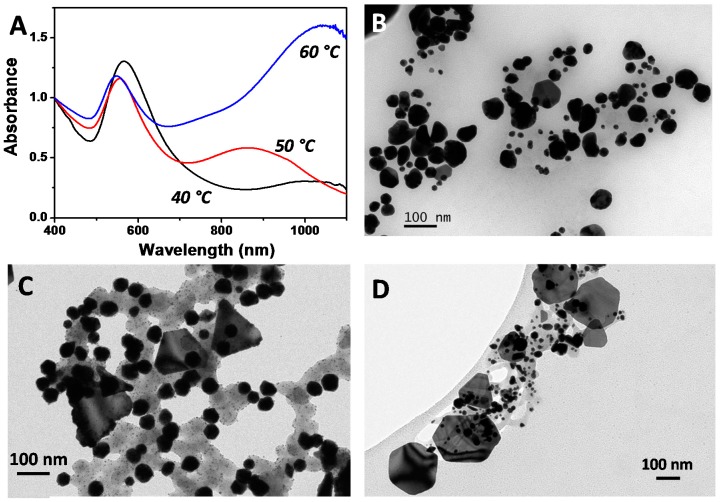
(**A**) Normalized Vis-NIR spectra of Au NPs obtained in the presence of the biosurfactant extracted from corn steep liquor at three different temperatures as indicated. (**B**–**D**) Representative TEM images of Au NPs obtained at 40 °C (**B**); 50 °C (**C**), and 60 °C (**D**).

There are several important facts to take into account in order to understand the process: at room temperature the mixture of salt precursor and biosurfactant remains colorless, even after several days; no additional chemicals are present in the reaction mixture; and the resulting Au NPs are stable for months. These facts allow us to postulate that the reducing power of the biosurfactant is limited, being induced by the temperature and, also the biosurfactant should act as stabilizing agent. Therefore, the temperature is a key parameter, which promotes the Au^+^ to Au^0^ reduction and can influence the nucleation and growth of Au NPs. Moreover, the separation of the nucleation and growth processes should give rise to more monodisperse particles. To investigate the influence of the temperature on the fabrication of Au NPs using the biosurfactant extracted from CSL, we have performed a series of experiments where the temperature of the mixture was kept at 40 °C, 50 °C and 60 °C. Figure 3A shows the Vis-NIR spectra of Au NPs obtained at different temperatures. The extinction spectra show very different optical features. Independently of the temperature, the resulting Au colloids exhibit two different LSPR bands, whose origin can be found in the spherical NPs (a dipole mode around 540 nm) and in the nanoplates (an in-plane dipole LSPR at longer wavelengths). Nevertheless, while at 40 °C the main band is that corresponding to nanospheres (the black spectrum in Figure 3A), as the temperature increases the LSPR generated by nanoplates get more intense. While the reaction performed at 50 °C lead to the appearance of a second band located at ca. 850 nm (red spectrum), a further increase of the reaction temperature to 60 °C produces a red shift in the position of the LSPR up to 1050 nm as well as an increase in its intensity (blue spectrum).

In order to confirm that, we performed TEM characterization of the different samples. Figure 3B–D shows representative TEM images of the AuNPs obtained in the presence of the biosurfactant at 40 °C, 50 °C and 60 °C, respectively. TEM analysis reveals that the sample synthesized at 40 °C is mainly composed of spherical NPs with an average diameter of 58.4 ± 12.8 nm, although a small population of nanoplates (3% based on TEM analysis) can be also observed (see Figure 3B and Figures S1–S3 in the supporting information). In the case of samples prepared at 50 °C and 60 °C, two well-defined populations can also be distinguished: spheres and plates. The population of plates increases with the temperature from 12% at 50 °C to 30% at 60 °C. It should be pointed out that at 60 °C, the optical response of the colloidal dispersion is dominated by the optical contribution of Au nanoplates, despite constituting just 30% of the total population. As shown in Figure 2A and Figure 3A, the main optical feature corresponds to the in-plane dipole LSPR of nanoplates. Moreover, the average size of the spherical nanoparticles is 49.9 ± 2.9 nm and 32.7 ± 8.5 nm for synthesis at 50 °C and 60 °C, respectively. Interestingly, the increase in the reaction temperature from 40 °C to 60 °C leads to a decrease in the average diameter of the particles from 58.4 to 32.7 nm. The decrease in nanoparticle size could be ascribed to the fact that lowering the reaction temperature decreases the reduction capabilities of the biosurfactant, leading to a lower number of nucleation events while promoting the growth on the initially-formed particles. Unfortunately, experiments at temperatures higher than 60 °C could not be performed, due to the structure loss of the biosurfactant. 

Likewise, the as-prepared NPs are negatively charged (zeta potential of −30.1 ± 1.3 mV), indicating that biosurfactant stabilization could be produced through the preferential adsorption of long chain carboxylic acids present in the biosurfactant composition [40]. 

In biosynthesis processes, nanoparticle formation, size and morphology rely on the biosurfactant composition. Thus, gold salt reduction and nucleation/growth processes could be ascribed to the presence of different components in the biosurfactant composition such as proteins, sugars or unsaturated fatty acids (linolelaidic acid and oleic or elaidic acid). In fact these components have been previously reported to act as reducing agents of different metal salts. For instance, unsaturated fatty acids have been recently used as the reducing agents to fabricate gold octahedral and triangular NPs [41,42,43]. Moreover, different proteins, such as human serum albumin (HSA), bovine serum albumin (BSA) or subtilisin Carlsberg (SC), have been also employed in the synthesis of gold NPs [44,45]. Alternatively, biosynthesis of Au NPs has been carried out using different sugars [14,46]. 

### 2.3. Synthesis of Ag NPs Using the Biosurfactant Extracted from CSL

The strategy for the preparation of Ag NPs mediated by the biosurfactant extracted from CSL is similar to that described for Au NPs. As in the case of Au NPs, the reaction was followed by UV-Vis-NIR spectroscopy. The initial mixture (Ag salt and biosurfactant solution) is colorless and subsequently evolves into a dark-yellow brown. After 3 h the spectrum remained unchanged, indicating the end of the reaction. It should be pointed out that at room temperature no reduction takes place, since the initial mixture solution remained colorless (data not shown). The nucleation and growth of silver NPs are only induced by the temperature. At 60 °C silver ions can be reduced by linolelaidic acid and oleic/or elaidic acid, which are present in the biosurfactant components. Both acids have vinyl groups in the carbon chain that can be oxidized with the temperature, allowing the reduction of Ag^+^ to Ag^0^, producing Ag NPs. 

Furthermore, considering the optical properties of the Ag NPs remained unchanged with time, and since no additional stabilizer or capping agent is present in the reaction medium, we postulate that the biosurfactant also acts as a stabilizing agent being adsorbed to the Ag NPs surface. The Ag NPs are also negatively charged (zeta potential of −31.0 ± 1.1 mV).

**Figure 4:**
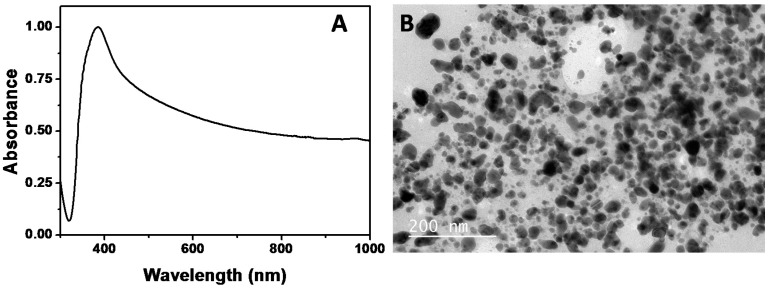
(**A**) Normalized UV-Vis-NIR spectrum of biosurfactant stabilized Ag NPs obtained by the reduction of silver nitrate at 60 °C; (**B**) Representative TEM image of the BS-Ag NPs.

Figure 4A shows the extinction spectrum of Ag NPs prepared using the CSL biosurfactant (BS-Ag NPs). A broad band centered on 400 nm dominates the optical properties of the BS-Ag NPs. The TEM analysis reveals that the sample is mainly composed of spherical NPs, with a broad size distribution between 5 and 60 nm (see a size distribution histogram in Figure S4, supporting information). It is well known that for one-step biocompatible synthesis, it is common to obtain non-uniform NPs (in size), since nucleation and growth take place mostly at the same time [47]. 

### 2.4. Antimicrobial Assay of Biosurfactant-Stabilized Ag NPs

Ag NPs obtained by green processes are highly compatible for pharmaceutical or other biomedical applications, their use as antimicrobial agents being one of the most important [48]. Although different works have reported the synthesis of Ag NPs using biosurfactants, many of these biosurfactants are produced by pathogenic microorganisms, preventing their application at an industrial scale [28,29]. The biosurfactant obtained from CSL is a biocompatible and natural surfactant, which acts as a reducing agent and stabilizer in the synthesis of Au and Ag NPs, as demonstrated in this work. Moreover, to the best of our knowledge, this is the first work where a biosurfactant extracted from corn steep liquor is employed to induce the synthesis of noble metal NPs.

Therefore, the next step was to analyze the antimicrobial activity of the synthesized BS-Ag NPs. In order to take into account the influence of the lipopeptide biosurfactant, we have also performed the study with Ag NPs stabilized by citrate ions (Ag@citrate NPs) of 15.5 ± 1.7 nm in diameter. Citrate stabilized NPs were synthesized according to previously reported procedures (see in SI and Materials and Methods section). Figure S5, in the supporting information, shows the UV-Vis-NIR spectrum, as well as a representative TEM image of the citrate stabilized Ag NPs.

The antimicrobial properties of Ag NPs, even at small doses, are well described in the literature [49,50,51,52]. Although the exact mechanism of cyto- and genotoxicity is not fully known, different mechanisms have been proposed. For instance, Hsueh et al. proposed that the antibacterial properties are due to the release of Ag^+^ ions from Ag NPs. These ions can penetrate into the bacterial cells, triggering the production of reactive oxygen species (ROS) species and causing a set of chromosomal aberrations and DNA damage. Another proposed mechanism is based on the specific interaction of Ag NPs with sulfur and phosphorous moieties present in the cell membrane, resulting in failure of metabolism and thereby leading to apoptosis/lysis of bacteria [53]. 

Ag NPs are protected by stabilizing agents that could also affect their antibacterial properties. For instance the cytotoxicity of some surfactants, like CTAB, increases the antibacterial activity of some NPs [54]. In this work, the CSL surfactant is harmless to bacterial microorganisms, as previously described, and therefore should not affect the antibacterial properties of the Ag NPs.

**Figure 5:**
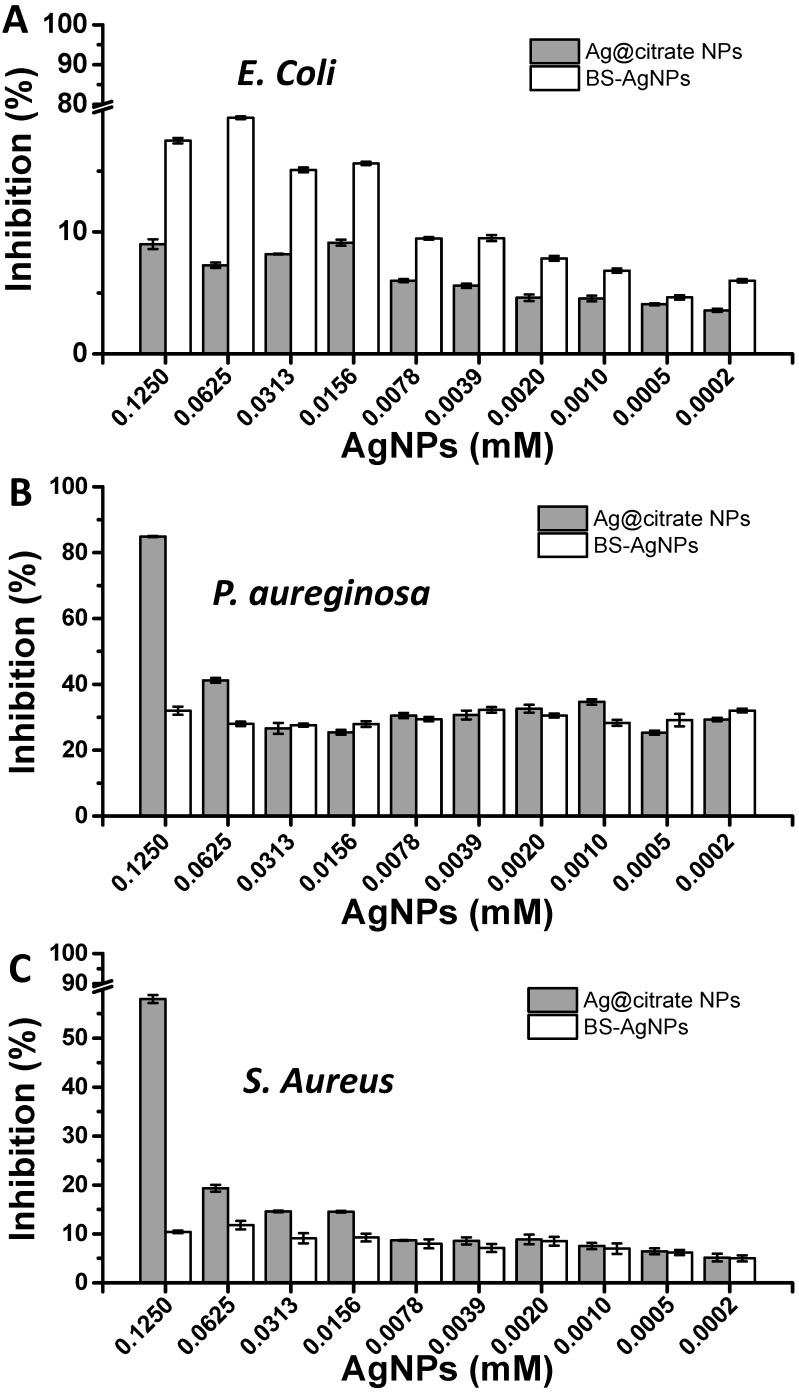
Inhibition percentages obtained with Ag@citrate NPs and BS-Ag NPs against (**A**) *E. coli* (**B**) *P. aeruginosa* and (**C**) *S. aureus* at different concentrations after 24 h. Results are averages of triplicate experiments.

In order to test the antimicrobial activity of BS-Ag NPs, three microorganisms (*E. coli*, *P. aeruginosa* and *S. aureus*) were grown in the presence of TSB medium and silver colloids (BS-Ag NPs or Ag@citrate NPs, the latter used as reference). Figure 5 shows the inhibition percentages achieved with *E. coli* (gram-negative bacteria), *P. aeruginosa* (gram-negative bacteria), and *S. aureus* (gram-positive bacteria) and induced by BS-Ag NPs or Ag@citrate NPs. As shown in Figure 5, BS-Ag NPs exhibits higher antimicrobial activity against gram-negative bacteria, like *E. coli* and *P. aeruginosa*, over the gram-positive bacteria, *S. aureus*. The antimicrobial activity difference between gram-negative and gram-positive bacteria is probably due to the electrostatic attraction between positively charged Ag NPs and negatively charged bacterial cells [55]. Moreover, Feng and co-workers [56] suggested that the cell walls of gram-positive bacteria have a peptidoglycan layer (sugars and amino acids structure) much thicker than that in gram-negative ones. For that, a higher concentration of BS-Ag NPs will be necessary for good antimicrobial activity against *S. aureus.*

Recently, Dhand and collaborators [47] found that Ag NPs obtained from dried roasted arabica coffee showed strong antimicrobial activity—comparable to that for ampicillin—against two different gram stained bacteria (*E. coli* and *S. aureus*). Although their results are in agreement with the data obtained in the present work, the concentration of Ag NPs used in the present work was 100 times lower than that used by Dhand and co-workers. Venkatpurwar and Pokharkar observed greater antibacterial activity against *E. coli*, as compared to *S. aureus* for Ag NPs produced by sulfated polysaccharide isolated from marine red algae [57]. 

In the case of Ag@citrate NPs, an important antimicrobial activity was observed at the highest concentration assayed (0.125 mM), being the highest against *P. aeruginosa* (84.9 ± 0.11%). Nevertheless, BS-Ag NPs show a lower inhibition effect against *P. aeruginosa*. Some authors have reported that the presence of biosurfactants can increase the resistance to some pathogenic microorganisms. For instance, Xie et al. [58] have reported that the presence of rhamnolipid increased the resistance of *E. coli* to Ag NPs. Therefore, this fact can be related to the higher biocompatible character of these NPs in comparison with the NPs obtained in the absence of biosurfactants.

Interestingly, it can be observed that BS-Ag NPs present a higher percentage of inhibition of *E. coli* (17.8 ± 0.22%) than Ag@citrate NPs (9.8 ± 0.40%). Although in both cases the percentage of inhibition was relatively low, probably due to the low concentration of Ag^0^, which is oxidized to Ag^+^. The Ag cations are responsible for the inhibition of the *E.* coli [49]. Although the inhibition percentages achieved by BS-Ag NPs are relatively low when compared to the inhibition percentages reported in the literature, it should be pointed out that we performed the experiments using lower amounts of silver. The highest Ag concentration used in our experiments was 0.125 mM, while most of the inhibition studies reported referred to Ag concentrations higher than 0.5 mM [48], meaning that BS-Ag NPs can be considered as quite effective in terms of inhibition. In this sense, further increase of BS-Ag NP concentration could improve the inhibition percentages achieved herein. Finally, the fact that BS-Ag NPs were produced by means of one-step green synthesis using an accessible, sustainable, biocompatible natural product offers them great potential for various bioapplications that are currently being investigated.

## 3. Materials and Methods 

### 3.1. Chemicals

Silver nitrate (AgNO_3_), gold (III) chloride trihydrate (HAuCl_4_·3H_2_O), sodium citrate (Na_3_C_6_H_5_O_7_), sodium borohydride (NaBH_4_), and corn steep liquor (CSL, liquid brown, 50% solid content and pH 4.4) were purchased from Sigma-Aldrich (Madrid, Spain). All chemicals were used as received.

### 3.2. Characterization Techniques

The optical characterization was carried out with a Cary 5000 UV-Vis-NIR spectrophotometer (Agilent techologies, Santa Clara, CA, USA) using 0.5 cm path length quartz cuvettes. Transmission electron microscopy (TEM) images were obtained with a JEOL JEM 1010 transmission electron microscope operating (JEOL Ltd., Tokyo, Japan) at an acceleration voltage of 100 kV.

### 3.3. Extraction and Characterization of Biosurfactant from CSL

The biosurfactant (BS) extraction was carried out using a liquid-liquid extraction process, with a CSL: organic phase ratio of 1:2 (*v*/*v*), at 150 rpm, for 1 h and at a temperature of 56 °C, following the protocol established by Vecino et al. [30]. Once the biosurfactant was extracted, it was dissolved in the same volume of water present at the beginning of the extraction process. Once dissolved in water, the biosurfactant was filtered by a 0.45 μm filter. The following aqueous solution, containing the biosurfactant, was diluted to reach a concentration two times higher than its critical micelle concentration (CMC).

The CMC of the biosurfactant extract obtained from CSL was determined by diluting the extract in water, and the surface tension of the solutions was measured by the Wilhelmy plate method in a force tensiometer with a platinum plate (Easy Dyne K20, KRUSS GmbH) at room temperature. All determinations were carried out in triplicate.

Additionally, in order to corroborate that the biosurfactant extracted from corn steep liquor was the same as that reported by Vecino et al. [30,31], Fourier transform infrared spectroscopy (FTIR) was carried out. Thus, 1 mg of biosurfactant was ground with 10 mg of potassium bromide and pressed (7500 kg for 30 s) to produce translucent pellets. FTIR analyses were carried out in a, Niocolet 6700 FTIR spectrometer (Thermo Scientific, Whaltham, MA, USA). The spectral measurements were made in the transmittance mode in a range of 400–4000 cm^−1^, with a resolution of 4 cm^−1^, an average data scanning range of 32, and a potassium bromide pellet was used to measure the background absorbance levels.

Moreover, the fatty acid composition of the biosurfactant was analyzed by gas chromatography (Trace GC Ultra, Thermo Scientific, Whaltham, MA, USA) coupled to a mass spectrometer (Trace DSQ, Thermo Finnigan, Waltham, MA, USA) after methylation and trans-esterification of fatty acids into fatty acid methyl esters (FAMEs) according to the method described previously [30]. FAMEs separation was performed on a ZB-WAX column (60 m × 0.25 mm i.d. × 0.25 μm film thickness) with an oven temperature gradient of 60 °C for 2 min, then 60–200 °C at 10 °C min^−1^, held for 27 min, then increased to 240 °C at 5 °C min^−1^ and finally held for 20 min. Helium was used as carrier gas at a flow rate of 1 mL min^−1^ and the temperature of both injector inlet and the transfer line of the detector were set at 250 °C.

The mass spectra were obtained using a mass selective detector under electron impact ionization at a voltage of 70 eV, and data were acquired over an *m*/*z* range of 40–400. FAMEs were identified from a mass spectra library supplied with the GC-MS system and by comparison of retention times and mass spectra of a FAME standard mix, injected under the same conditions.

### 3.4. Synthesis of Au NPs

Au NPs were synthesized by injecting a solution of gold chloride trihydrate under magnetic stirring in 5 mL stock solution of biosurfactant. The final concentration of gold was 0.25 mM. Immediately after the gold addition, the temperature of the mixture was heated to a certain temperature (40 °C, 50 °C or 60 °C, depending on the experiment). The reduction of gold salt can be followed by naked eye due to the color change observed, from light yellow, colorless to red indicating the Au NP formation. An aliquot of the obtained samples were taken to perform the UV-Vis-NIR characterization as well as the TEM.

### 3.5. Synthesis of Ag NPs

Two different syntheses of Ag NPs were performed: citrate stabilized Ag NPs (Ag@citrate NPs), and biosurfactant stabilized Ag NPs (BS-Ag NPs). For the first synthesis (Ag@citrate NPs), 5 mL of silver nitrate (0.5 mM) was mixed with 5 mL of sodium citrate (10 mM). Subsequently, 100 µL of sodium borohydride (0.1 M) was quickly added under magnetic stirring [59]. 

For the synthesis of BS-Ag NPs, the process is similar to that previously described for Au NPs. Briefly, an aqueous solution of silver nitrate was added under magnetic stirring to a pure solution of biosurfactant. The final concentration of silver was 0.25 mM. Afterwards the temperature of the mixture was heated to 60 °C. The reduction process of silver from Ag^+^ to Ag^0^ takes place when the temperature is increased. The reduction of silver can be followed due to the color change observed, from colorless to dark brown, indicating that the reaction has finished. An aliquot of the obtained sample was taken to perform the UV-Vis-NIR characterization as well as the TEM. The rest was used to perform the antimicrobial assays.

### 3.6. Antimicrobial Assay 

*Escherichia coli* (*E. coli*), *Pseudomona aeruginosa* (*P. aeruginosa*) and *Staphylococcus aureus* (*S. aureus*) were used for antimicrobial assay. These strains were cultivated in Trypticase Soy Broth (TSB) (OXOID, Basingstoke, UK) at 37 °C, for 24 h at 180 rpm.

The antimicrobial activity of Ag@citrate NPs and BS-Ag NPs against microbial strains was determined according to the procedure previously described by Gudiña et al. [60], using a micro-dilution method in 96-well flat-bottomed plastic tissue culture plates (Greiner Bio-One GmbH, Frickenhausen, Germany). In the 1 column of the 96-well micro-plate were placed 125 μL of sterile double strength medium (TSB), while 125 μL of sterile single strength growth medium was placed in the remaining wells (2–12 columns). Consequently, 125 μL of Ag NPs synthesized with biosurfactant, or Ag@citrate NPs (initial silver concentration of 0.25 mM), were added to the 1 column of the micro-plate and mixed with the medium. Serially, 125 μL were transferred to the subsequent wells, discarding 125 μL of the mixture in the tenth column, so that the final volume for each well was 125 μL.

Columns 11 and 12 did not contain Ag@citrate NPs or BS-Ag NPs solution, and served as growth and negative controls, respectively. All wells (except for column 12) were inoculated with 2.5 μL of a pre-culture of the corresponding microorganism grown overnight in TSB medium at 37 °C and diluted to an optical density (600 nm) of 0.6.

The micro-plates were covered, incubated for 24 h at 37 °C, and the optical density of each well (at 600 nm) was measured after this time. The growth inhibition percentages at different Ag@citrate NPs or BS-Ag NPs concentrations for each microorganism were calculated as:

(1) $$Growth\;inhibition_{c}\left(\%\right)=\left[1-\frac{\left(OD_{c}\right)}{\left(OD_{0}\right)}\right]\;\times \;100$$

where *OD_c_* represents the optical density of the well with different Ag@citrate NPs or BS-Ag NPs concentrations, and *OD*_0_ is the optical density of the control well (without nanoparticle solution). Triplicate assays were performed at all the Ag@citrate NPs or BS-Ag NPs concentrations for each strain. 

## 4. Conclusions

In this work, we have proposed a green synthesis protocol to obtain Ag and Au NPs in a single-step process mediated by a natural biosurfactant extracted from CSL. Although the biosurfactant presents limited reduction capability at room temperature, it can be improved by increasing the temperature to 60 °C. While in the case of Ag, mainly nanospheres are obtained, the reduction of a gold precursor gives rise to a mixture of nanospheres and nanoplates. Moreover, the temperature promotes the formation of nanoplates, which present the LSPR in the NIR region, dominating the optical response of the colloidal dispersion. The dual functionality of the biosurfactant, acting as reducing agent as well as stabilizer, makes it particularly useful for green synthesis approaches. Since the biosurfactant was obtained from a GRAS microorganism, the synthesized NPs could be considered as biocompatible. Furthermore, the preformed biocompatible BS-Ag NPs were used in antimicrobial tests, exhibiting a high antimicrobial activity, against gram-negative bacteria like *E. coli*, at a very low concentration of silver.

## Figures and Tables

**Table 1:** Fatty acid composition of the biosurfactant extracted from corn steep liquor (CSL) [31].

Fatty Acid	Formula	Rel. Abundance (%)
Palmitic acid	C_16_H_32_O_2_	22.0 ± 2.2
Stearic acid	C_18_H_36_O_2_	6.4 ± 1.4
Oleic or elaidic acid	C_18_H_34_O_2_	22.5 ± 1.8
Linolelaidic acid	C_18_H_32_O_2_	45.9 ± 6.4
Palmitic acid	C_16_H_32_O_2_	22.0 ± 2.2

## Supplementary Materials

The following are available online at http://www.mdpi.com/2079-4991/7/6/139/s1, Figures S1–S5.
