# Supplementary material for: Biogenic Synthesis of Metal Nanoparticles Using a Biosurfactant Extracted from Corn and Their Antimicrobial Properties
Source: Nanomaterials (Basel). 2017 Jun 6;7(6):139. doi: 10.3390/nano7060139 (PMC5485786; doi:10.3390/nano7060139)
Supplement: Supplementary file 1 [file nanomaterials-07-00139-s001.pdf]

Supporting information

for

Biogenic Synthesis of Metal Nanoparticles Using a Biosurfactant Extracted from Corn and Their Antimicrobial Properties

Sergio Gómez-Graña,[1,*] María Perez-Ameneiro,[2] Xanel Vecino,[2,3] Isabel Pastoriza-Santos,[1] Jorge Perez-Juste,[1] Jose Manuel Cruz,[2] Ana Belén Moldes[2]

[1]Departamento de Química Física y CINBIO, Universidade de Vigo. 36310, Vigo, Spain.

[2]Chemical Engineering Department, School of Industrial Engineering (EEI), University of Vigo. Campus As Lagoas-Marcosende. 36310 Vigo-Pontevedra, Spain.

[3]CEB-Centre of Biological Engineering, University of Minho, Campus de Gualtar. 4710-057 Braga, Portugal.

[*] corresponding author: segomez@uvigo.es

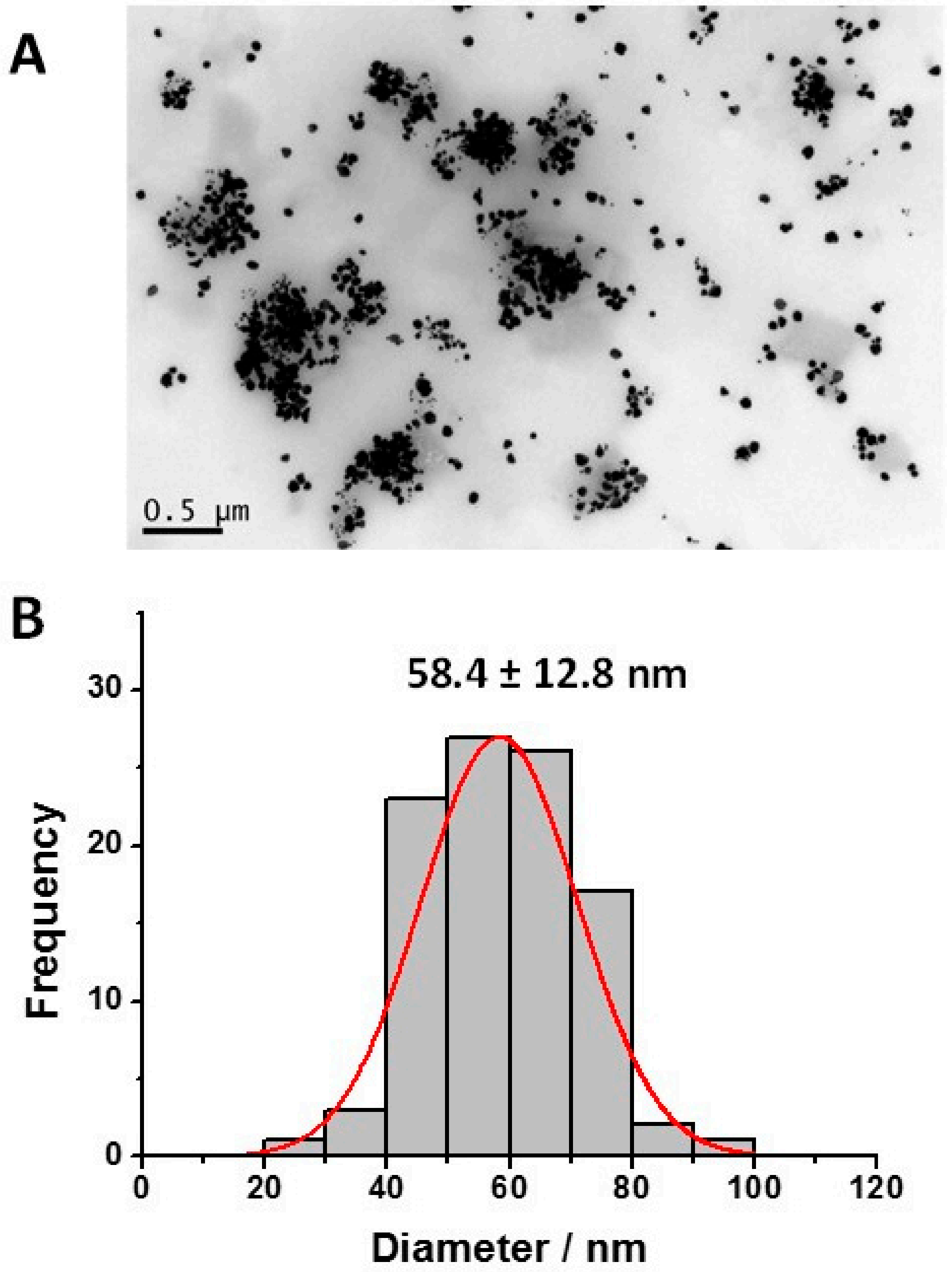

Figure S1. (A) Representative TEM image of Au NPs synthesized in the presence of biosurfactant extracted from CSL and its corresponding size distribution histograms for the spherical Au NPs synthetized at 40°C (B).

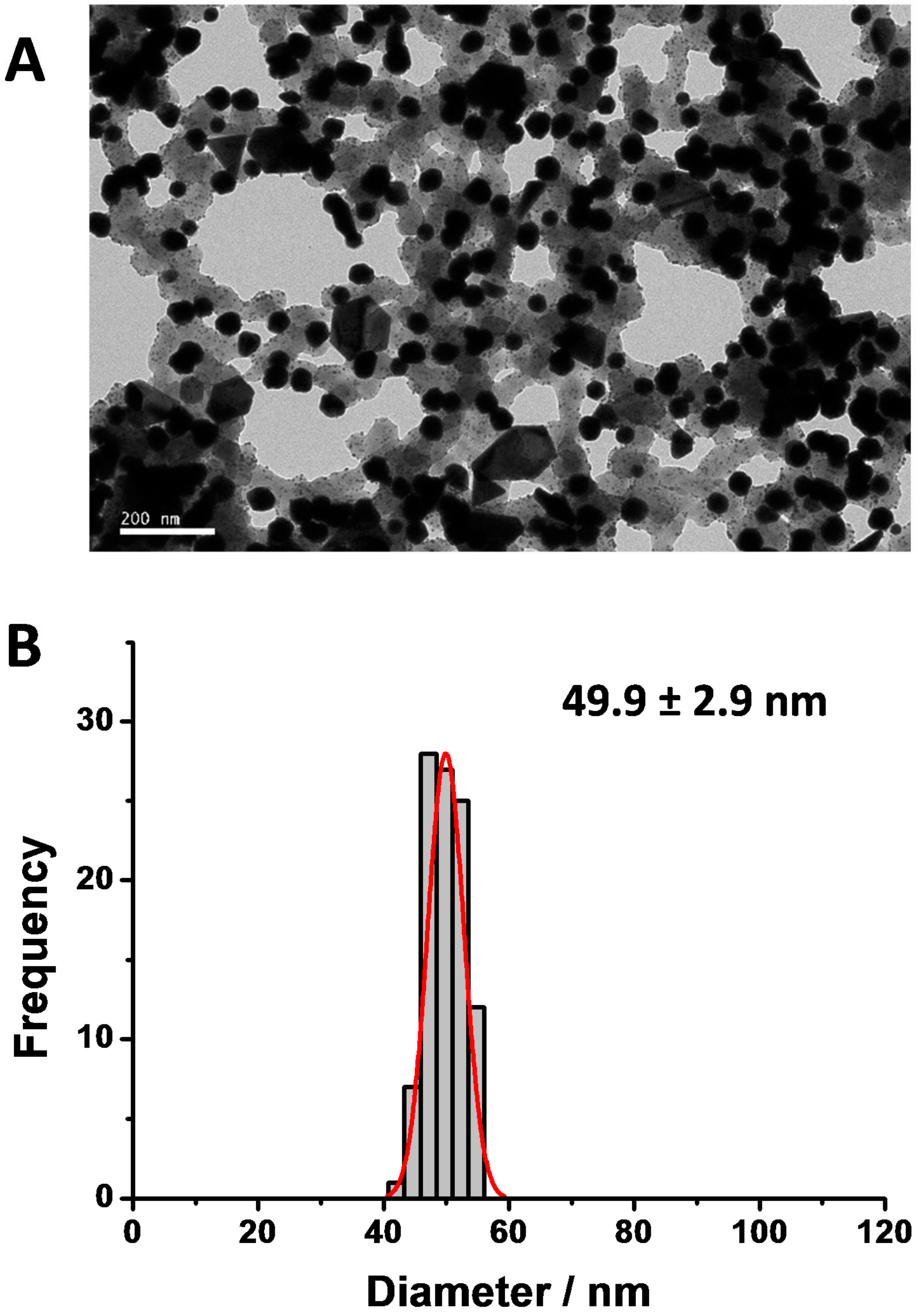

Figure S2. (A) Representative TEM image of Au NPs synthesized in the presence of biosurfactant extracted from CSL and its corresponding size distribution histograms for the spherical Au NPs synthetized at 50°C (B).

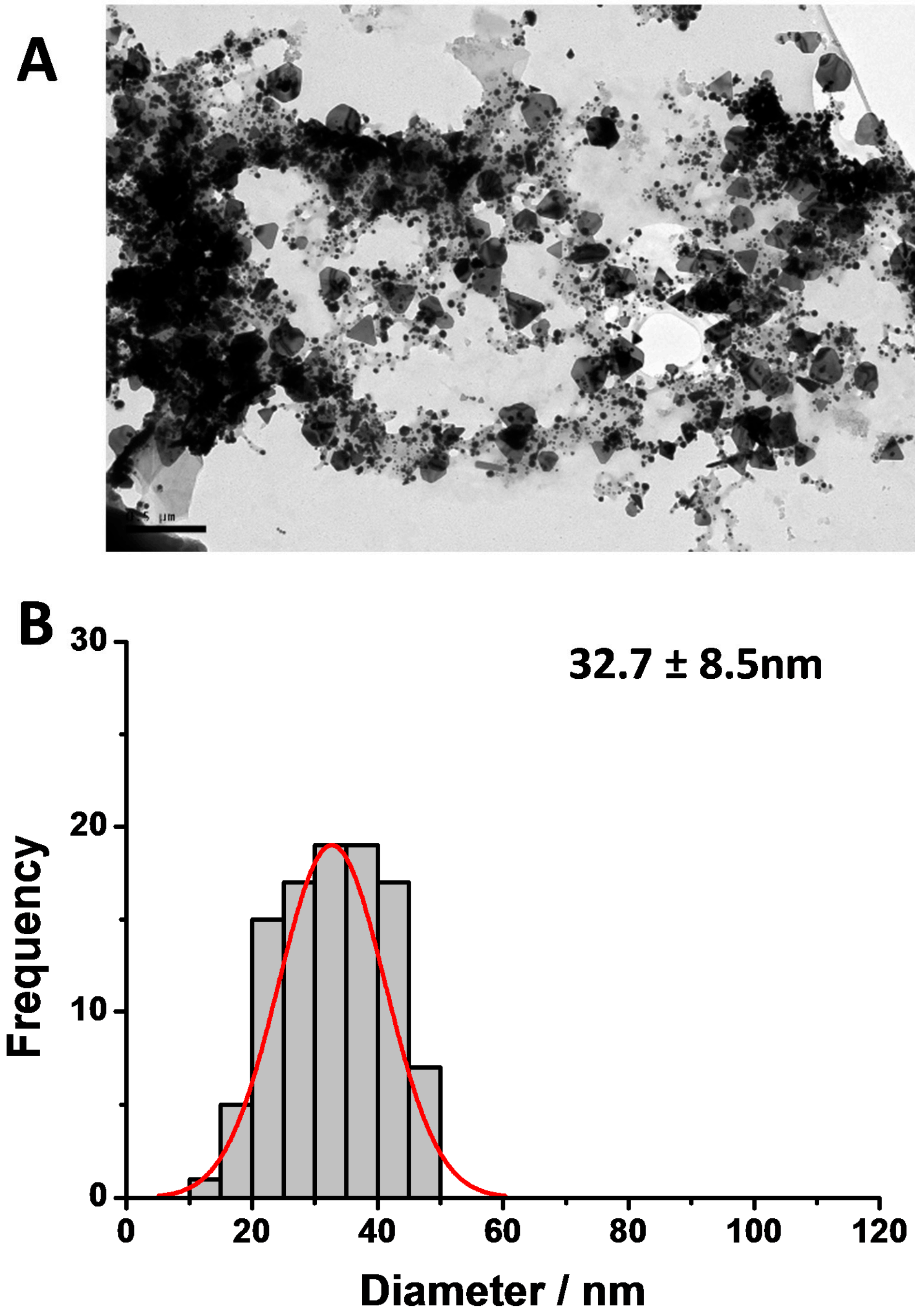

Figure S3. (A) Representative TEM image of Au NPs synthesized in the presence of biosurfactant extracted from CSL and its corresponding size distribution histograms for the spherical Au NPs synthetized at 60°C (B).

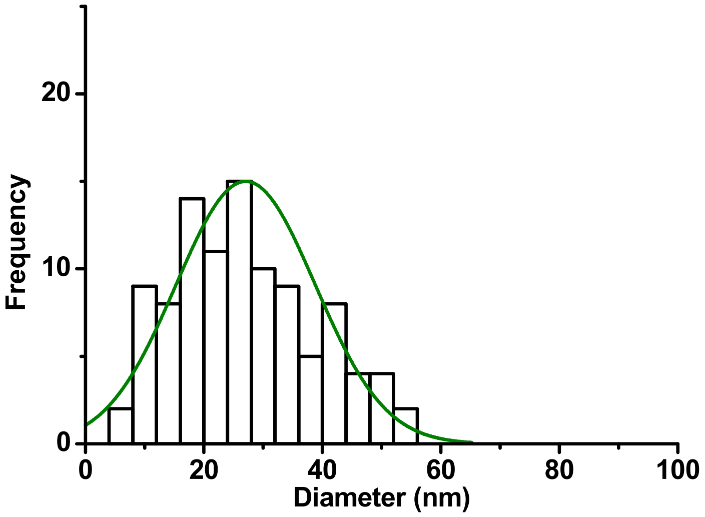

Figure S4. Size distribution histogram for the Ag NPs synthesized in the presence of the biosurfatant.

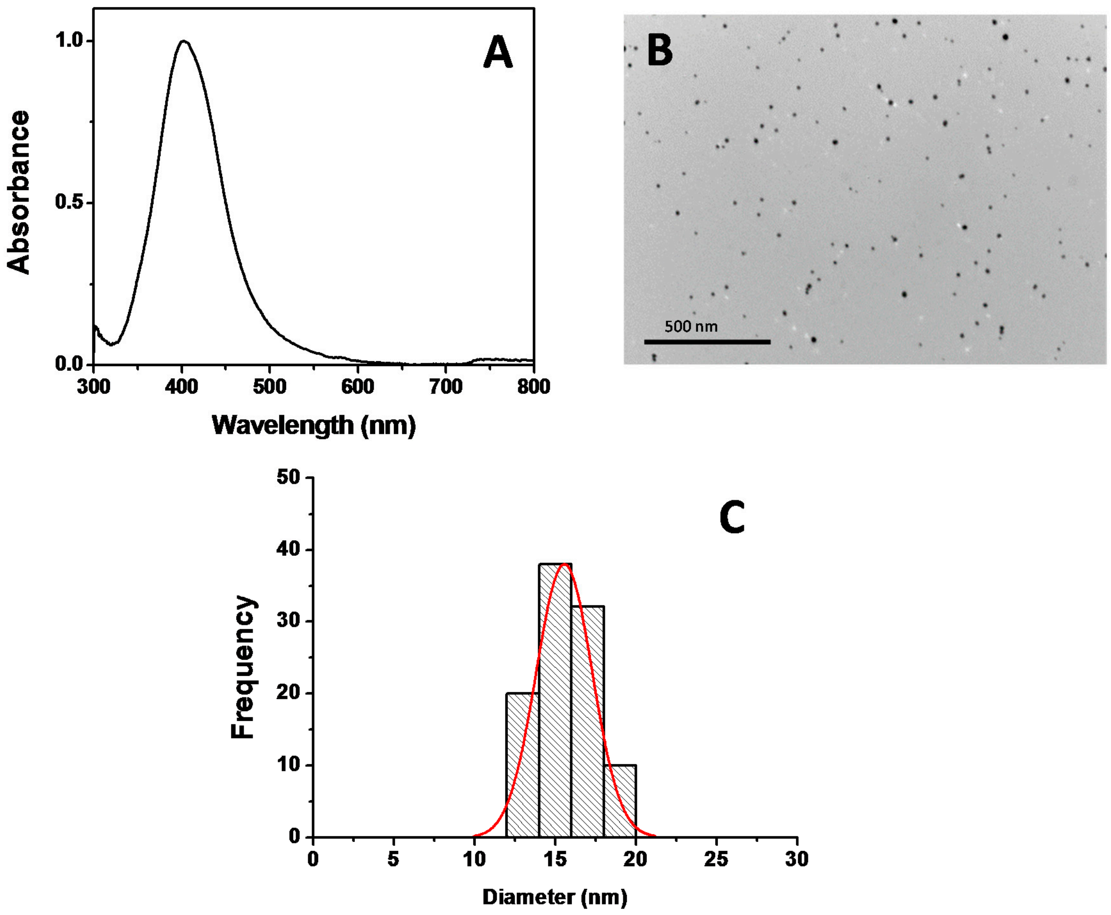

Figure S5. (A) UV-Vis spectrum of a dispersion of citrate stabilized Ag NPs. (B) Representative TEM image of Ag@NPs synthesized in the presence of sodium citrate and their corresponding size distribution histograms (C).
